# Recent clinical trials inform the future for malaria vaccines

**DOI:** 10.1038/s43856-021-00030-2

**Published:** 2021-08-25

**Authors:** Liriye Kurtovic, Linda Reiling, D. Herbert Opi, James G. Beeson

**Affiliations:** 1 Burnet Institute, Melbourne, VIC Australia; 2 Department of Immunology and Pathology, Central Clinical School, Monash University, Melbourne, VIC Australia; 3 Department of Medicine, The University of Melbourne, Melbourne, VIC Australia; 4 Department of Microbiology, Monash University, Clayton, VIC Australia

**Keywords:** Vaccines, Malaria

## Abstract

Kurtovic et al. highlight some of the recent advances in the development and clinical evaluation of malaria vaccines. The authors outline key vaccine strategies and clinical trials, and discuss priorities for research into the development of an efficacious malaria vaccine.

## Teaser

Malaria vaccines are urgently needed in the fight against this devastating disease that is responsible for almost half a million deaths each year. Here, we discuss recent clinical advances in vaccine development and highlight ongoing challenges for the future.

Malaria is one of the most devastating infectious diseases in humans, responsible for >200 million cases and almost half a million deaths annually. Control programs have significantly reduced disease burden since 2000, but numbers have plateaued since 2015. The disruption caused by the ongoing COVID-19 pandemic will further unwind these efforts and is predicted to increase the number of malaria deaths. New control measures are urgently needed, and lessons learned from emerging diseases such as COVID-19 highlight the enormous potential of effective vaccines. This has been recognized by the World Health Organization (WHO) and key stakeholders who recommend that, within the next decade, a vaccine should be developed that is at least 75% efficacious against malaria over 2 years (i.e., reduces disease occurrence by at least 75% in vaccinated compared to unvaccinated individuals) and also reduces malaria transmission.

This is an exciting time for malaria vaccine development, with a number of promising clinical trials recently completed and others currently underway…

**Fig. 1:**
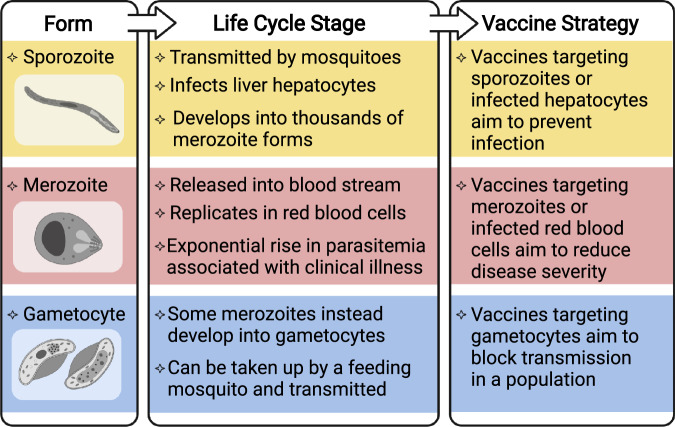
Vaccine strategies against different life cycle stages of *Plasmodium spp*. Overview of the morphologically distinct sporozoite, merozoite, and gametocyte forms at different stages of the parasite life cycle, and the vaccine strategy for targeting each stage. Vaccines that target multiple life cycle stages are also in development. Created with BioRender.com.

*Plasmodium falciparum* causes the majority of malaria burden and has been the primary focus of vaccine development, and *Plasmodium* *vivax* is the second major cause of malaria. There are several vaccine strategies that target different developmental stages of the malaria-causing parasite (Fig. 1). This begins with the sporozoite parasite form, which is inoculated into the skin by a feeding mosquito, circulates in the blood, and infects the liver to develop into the morphologically distinct merozoite form. Merozoites then replicate within red blood cells to exponentially increase parasitemia and can cause severe disease pathology. Some merozoites will undergo development into the gametocyte form that can be taken up by the mosquito vector and subsequently be transmitted in a population. An important bottleneck in the vaccine development pipeline is the assessment of potential candidates in clinical trials. Given the limitations of malaria animal models to adequately reflect human disease, the controlled human malaria infection (CHMI) model offers a unique ability to fast-track initial evaluations of vaccine efficacy. Safe and controlled parasite infection in a clinical setting has proven more time-efficient and less variable than natural parasite exposure and can therefore rely on fewer subjects for conducting the first efficacy trial with a vaccine. CHMI has also been increasingly implemented in malaria-endemic settings, which is critically important to understand the dynamics between vaccine-induced immunity and pre-existing immunity during ongoing natural exposure to malaria.

Here, we discuss recent advances and insights from clinical trials of malaria vaccines and highlight ongoing challenges and priorities for the future. Table 1 lists the key trials discussed in this Comment.

**Table 1:** Summary of recent and ongoing malaria vaccine clinical trials.

Vaccine	Dosage	Phase; trial no.	Population	Outcome; follow-up	Ref.
*Vaccines targeting sporozoites*					
PfSPZ	3 doses of 9×10^5^	1; NCT02613520	18–45 years, Tanzania (*n* = 5)	100% efficacy against CHMI at 23–79 days	^1^
	3 doses of 9 × 10^5^	1; NCT02687373	5–12 months, Kenya (*n* = 8)	Safe and immunogenic over 29 days	^2^
PfSPZ-CVac	2 doses of 2 × 10^5^ with chloroquine	1; NCT03083847	Malaria-naive adults (*n* = 6)	100% efficacy against CHMI at 3 months	^3^
GAP3KO	Bites from 200 infected mosquitoes	1; NCT03168854	Malaria-naive adults (*n* = 16)	Vaccine efficacy pending (unpublished)	NA
RTS,S	3 doses of 25 µg	3; NCT00866619	5–17 months, multiple African sites (*n* = 4296)	56% efficacy against first or only malaria episode over 12 months	^4^
	2 doses of 50 µg, 1 delayed dose of 10 µg	2; NCT01857869	Malaria-naive adults (*n* = 30)	87% efficacy against CHMI at 3 weeks	^5^
	2 doses of 25 µg, 1 delayed dose of 5 µg	2; NCT03276962	5–17 months; Ghana and Kenya	Vaccine efficacy pending (unpublished)	NA
	RTS,S with seasonal malaria chemoprevention	3; NCT03143218	5–17 months; Burkina Faso and Mali	Vaccine efficacy pending (unpublished)	NA
R21	3 doses of 5 µg	2; NCT03896724	5–17 months; Burkina Faso (*n* = 146)	71–76% efficacy against at least one malaria episode over 12 months (depending on adjuvant dosage)	^6^
	3 doses of 5 µg	3; NCT04704830	5–36 months; multiple African sites	Vaccine efficacy pending (unpublished)	NA
*Vaccines targeting blood stages*					
RH5.1	3 doses of 10 µg	2; NCT02927145	Malaria-naive adults (*n* = 14)	1-day delay in parasitemia following CHMI at 14 days	^8^
	Dose escalation study	1; NCT04318002	5–17 months, 18–45 years, Tanzania	Vaccine safety and immunogenicity pending (unpublished)	NA
PAMVAC	3 doses of 50 µg	1; NCT02647489	Malaria-naive adults (*n* = 27)	Safe and immunogenic over 6 months	^11^
PRIMVAC	3 doses of 100 µg	1; NCT02658253	Burkina Faso (*n* = 20)	Safe and immunogenic over 35 days	^12^
*Transmission-blocking vaccines*					
Pfs230D1M	2 doses of 40 µg	1; NCT02334462	Malaria-naive adults (*n* = 5)	Safe and immunogenic over 56 days	^13^
	3 doses of 40 µg	2; NCT03917654	1 year and older; Mali	Vaccine efficacy pending (unpublished)	NA

Table summarizes the vaccine dosage, population, and outcomes from recent and ongoing clinical trials. Note that this is not a comprehensive list of all completed or ongoing malaria vaccine trials. Not all vaccine groups and results from each trial are included in the table. For full details, refer to the referenced publications. *NA* not available, *CHMI* controlled human malaria infection.

## Vaccines can block infection by targeting sporozoites

Vaccines can target sporozoites to prevent or impair infection of the liver, and therefore aim to induce immunity whereby an individual is protected against infection and disease. Live-attenuated sporozoite-based vaccines have the capacity to induce sterilizing immunity (no detectable parasitemia) against CHMI in malaria-naive volunteers. This includes the PfSPZ vaccine that contains radiation-attenuated sporozoites incapable of replicating. It was recently shown that an optimized PfSPZ vaccine dosage induced sterilizing immunity against CHMI in six Tanzanian adults^1^, and this dose appeared safe in African children, which supports further evaluations in younger age groups (NCT02687373)^2^. Another approach is to combine replication–intact sporozoites with antimalarial drug prophylaxis to prevent malarial illness, known as PfSPZ-CVac. PfSPZ-CVac can induce sterilizing immunity in malaria-naive volunteers, but there is no published data in malaria-endemic populations^3^. A third approach is to genetically attenuate sporozoites to prevent intrahepatic development. This includes the triple-gene knockout vaccine, GAP3KO, which recently completed an efficacy trial in malaria-naive volunteers (NCT03168854). Although sporozoite-based vaccines are promising, major obstacles include the fact that currently sporozoites cannot be cultured in vitro and are therefore isolated from infected mosquito salivary glands, and they currently require direct venous inoculation of vaccine doses and storage and transportation of vaccines using liquid nitrogen.

Subunit vaccines aim to target a key antigen, or sub-region, and have the advantage of using existing production technologies and delivery using established infrastructure and systems. The most successful is the RTS,S vaccine based on a truncated form of the major sporozoite surface antigen, circumsporozoite protein (CSP). This is expressed as a virus-like particle and administered with an adjuvant (AS01), which is used to enhance the immune response to vaccination. Promising findings from a phase 3 clinical trial in >15,000 African children led to pilot implementation of a four-dose booster regimen in children aged 5 months in Ghana, Kenya, and Malawi to further evaluate vaccine safety and efficacy, which is ongoing^4^. Additional trials are underway in African children to evaluate a modified regimen where a fraction of the third dose is administered months later, which appeared to be moderately more efficacious in malaria-naive volunteers (NCT03276962)^5^. There is also interest in whether RTS,S can synergize with seasonal malaria chemoprevention (NCT03143218).

A recent advance was the development of the R21 malaria vaccine, a virus-like particle that is similar to RTS,S, but has higher density of the CSP antigen on the particle surface and is formulated with Matrix-M adjuvant^6^. Initial results from a pediatric phase 2 clinical trial in Burkina Faso demonstrated 76% efficacy against malaria over 12 months^6^. Although this was considerably higher than vaccine efficacy achieved in previous RTS,S trials^4^, R21 was administered prior to the peak malaria season and most malaria occurred in the first 6 months^6^, making direct comparisons with RTS,S efficacy difficult to interpret. Furthermore, antibody levels had declined markedly by 12 months. Participants are still being monitored to determine vaccine efficacy over 2 years, and children are currently being enrolled in phase 3 clinical trials across multiple African sites (NCT04704830).

## Vaccines targeting blood stages of malaria prevent clinical illness

Blood-stage vaccines target the merozoite form or infected red blood cells and aim to control parasitemia and prevent clinical illness. There has been considerably less progress in achieving good efficacy for blood-stage vaccines, with only a handful of candidates evaluated in phase 2 clinical trials, and none having progressed further^7^. The most recent was RH5.1 comprised of full-length RH5 protein, an indispensable merozoite invasion ligand. In a phase 1/2a clinical trial in malaria-naive volunteers, RH5.1 with AS01 adjuvant was highly immunogenic but only resulted in a 1-day delay until threshold parasitemia levels were reached following CHMI compared with that of the unvaccinated control group^8^. An upcoming phase 1 clinical trial of RH5.1 will further evaluate safety and immunogenicity in Tanzanian adults and children (NCT04318002).

Merozoites express a multitude of surface proteins and invasion ligands, including many that are polymorphic, which makes it difficult to identify candidate antigens for subunit vaccines. In addition, there are major knowledge gaps on specific mechanisms that mediate protective antimalarial immunity. Antibodies play a critical role, but the current reference growth inhibition assay (GIA) that measures antibody inhibitory activity has neither strongly nor consistently been associated with protection against malaria^7^. Several vaccines, including RH5.1, have generated substantial GIA activity but shown limited efficacy^8^. Achieving higher efficacy with merozoite vaccines may require harnessing multiple immunologic mechanisms. Recent studies found that antibodies can activate complement and mediate Fcγ-receptor effector functions against merozoites, which were associated with clinical protection in malaria-endemic population studies^9^. This creates a new opportunity to evaluate the targets of functional antibody responses and potentially identify new vaccine candidate antigens using these novel approaches^9^.

Given the lack of success in developing blood-stage subunit vaccines, there is interest in whole parasite blood-stage vaccines, which present a large antigen repertoire to the immune system and may overcome the challenge of antigen-specific polymorphisms. The first clinical study of chemically attenuated parasite-infected red blood cells promisingly induced cellular immune responses in malaria-naive volunteers, but parasite-specific antibodies were not detected^10^. Further studies are needed on this particular vaccine approach, including improvements in vaccine production and formulation to avoid immune responses to the red blood cell membrane.

Once a merozoite infects a red blood cell and matures, the host cell undergoes extensive changes and expresses parasite-derived proteins on the red blood cell surface. One such protein, VAR2CSA, facilitates the accumulation and sequestration of parasite-infected red blood cells in the placenta. Malaria during pregnancy is, therefore, a major concern and can lead to adverse outcomes such as maternal anemia, preterm delivery, stillbirth, and low birthweight babies. Two VAR2CSA-based vaccines (PAMVAC and PRIMVAC) aimed at inhibiting placental malaria have recently completed phase 1 clinical trials and were immunogenic and had acceptable safety profiles, but are yet to be evaluated for vaccine efficacy^11,12^.

## Developing vaccines that block malaria transmission

Vaccines that target the gametocyte and gamete forms aim to prevent parasite transmission to the mosquito and thereby block malaria transmission throughout a population. Although this vaccine approach does not offer any direct clinical protection in humans, blocking malaria transmission is an essential component of effective control and elimination strategies. There has been limited progress for transmission-blocking vaccines, with one candidate based on the gametocyte surface antigen, Pfs230 (Pfs230D1M), showing promise in early clinical trials and a phase 2 clinical trial is ongoing in Mali (NCT03917654)^13^.

These vaccine approaches targeting different stages of the parasite life cycle have the ability to afford protection against infection, disease, and/or prevent parasite transmission among a population. Although each approach is of value, there may be additional benefits to developing multi-stage subunit vaccines that incorporate antigens expressed at different developmental stages.

## Future directions and opportunities

**Fig. 2:**
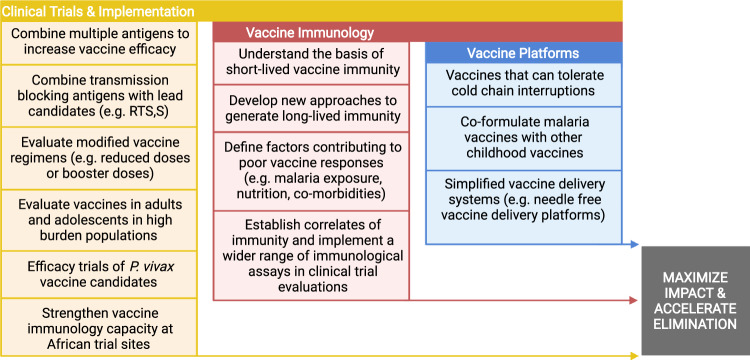
Priority research areas needed to further advance malaria vaccine development. The figure lists several research priorities, grouped into three main themes, for achieving malaria vaccines with high impact and have the potential to accelerate and sustain malaria elimination. Created with BioRender.com.

This is an exciting time for malaria vaccine development, with a number of promising clinical trials recently completed and others currently underway, although there are still considerable challenges that stand in the way of achieving vaccine goals within the next decade with several priority areas for research (Fig. 2). Malaria exposure may drive the lower vaccine responses typically observed in malaria-exposed populations compared with malaria-naive individuals. As disease burden can be substantial among adolescents and adults in some populations, clinical trials to evaluate vaccine efficacy in these groups will also be needed. A persisting challenge for malaria vaccines is achieving sustained protective efficacy as antibody and cellular responses are often short-lived and wane within a year^6^. The immunological factors underlying these vaccine limitations remain poorly understood but will be crucial for achieving higher efficacy and wider vaccine implementation in endemic populations. Furthermore, very little progress has been made toward the development of vaccines against *P. vivax*, which needs to be accelerated.

Recent advances in vaccine technologies, including those used in licensed COVID-19 vaccines, have opened new opportunities for malaria vaccine development. This includes mRNA-based vaccine platforms of the CSP malaria antigen, which has already shown encouraging results in animal models^14^. There has also been continued interest in heterologous prime-boost with ChAd43 and Modified Vaccinia Ankara viral vectors encoding different malaria antigens alone or in combinations (e.g., AMA1, MSP1, ME-TRAP)^15^.

Further research on these areas will greatly advance the development of effective malaria vaccines in endemic populations.
